# Comprehensive analysis of novel cubic HgCrO_3_ perovskite: a first principles, structural, thermodynamic, and magnetic properties study for spintronic applications

**DOI:** 10.1039/d3ra06392e

**Published:** 2023-11-16

**Authors:** Junaid Khan, Murefah mana Al-Anazy, El Sayed Yousef, Datta D, Ramesh Sharma, A. J. A. Moayad

**Affiliations:** a Department of Physics Khushal Khan Khattak University Karak Pakistan; b Department of Physics Kohat University of Science and Technology Kohat Pakistan; c Department of Chemistry, College of Sciences, Princess Nourah bint Abdulrahman University (PNU) P.O. Box 84428 Riyadh 11671 Saudi Arabia; d Research Center for Advanced Materials Science (RCAMS), King Khalid University Abha 61413 P. O. Box 9004 Saudi Arabia; e Physics Dep., Faculty of Science, King Khalid University P. O. Box 9004 Abha Saudi Arabia; f Dept. of Applied Science, Feroze Gandhi Institute of Engineering and Technology Raebareli 229001 Uttar Pradesh India sharmadft@gmail.com; g Department of Materials Science, Malawi University of Science and Technology, P. O. Box 5196 Limbe Malawi mailme_moya123@rediffmail.com

## Abstract

The main goal of modern manufacturing is to create products that are affordable, eco-friendly, and energy-efficient. With a focus on HgCrO_3_, this study sought to discover molecules that meet these requirements. The structural, electrical, thermodynamic, and transport properties of the material were investigated using Wien2K, a full-potential, linearized augmented plane wave program (FP LAPW). Utilizing the generalised gradient approximation (GGA) and lattice constants that have previously produced excellent theoretical and practical findings, structural optimization was carried out. Calculated HgCrO_3_ magnetic characteristics show that the Cr and Hg atoms are the main contributors to magnetism. Over a temperature range of 0–1200 K and a pressure range of 0–196 GPa, thermodynamic characteristics were evaluated. The thermoelectric properties of HgCrO_3_ were evaluated using the Boltzmann transport method provided by the BoltzTrap program. This analysis revealed that at room temperature, the figures of merit (*ZT*) values for HgCrO_3_ were nearly equal to one. A *ZT* value close to one indicates that a material has excellent thermoelectric properties and can efficiently convert heat into electricity or *vice versa*. This investigation highlights the promising thermoelectric capabilities of HgCrO_3_, which could contribute to more sustainable and energy-efficient technologies in the future.

## Abstract 2

The structural, electrical, thermodynamic, and transport properties of a cubic HgCrO_3_ perovskite were investigated using first principles computation. HgCrO_3_ showed good thermoelectric qualities that may potentially lead to more energy-efficient and sustainable technologies.

## Graphical abstract

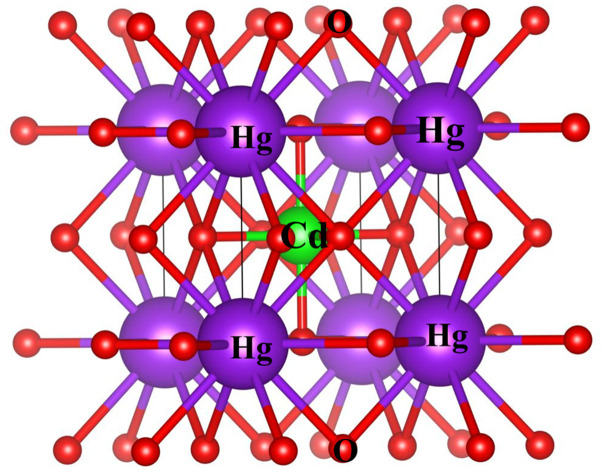

## 1. Introduction

Perovskite oxides, an inorganic substance, have attracted a lot of attention lately due to their exceptional abilities in the areas of electrical, structural, optical, thermoelectric, and elastic behaviours. These materials have a variety of crystalline phases and physicochemical characteristics, including ferroelectricity, ferro-elasticity, anti-ferroelectricity, and others.^1–4^ In the subject of spintronics, which studies ways to harness electron spin to store, process, and transfer information, ferromagnetic perovskite materials in particular have grown in importance. The flexible crystal structure of perovskite oxides is a key characteristic that allows for the development of materials with diverse electrical and other properties. To maintain the stability of the ABO_3_ compound structure in perovskites, a standard cubic ABO_3_ crystal structure is commonly employed. In this structure, ‘A’ and ‘B’ represent two different cations, ‘O’ stands for an anion, and the radius of ‘B’ is typically smaller than that of the ‘A-cation. In an ideal cubic ABO_3_ structure, an oxygen atom typically occupies the unit cell's centre. This arrangement also places a ‘B’ atom at the centre, with another atom often located at one of the cell's corners. This structural flexibility is crucial for the versatile properties of perovskite oxides, making them highly attractive for various applications, including in the field of materials science, where tailored properties are essential for technological advancements. The ability to fine-tune perovskite structures allows for the creation of materials with specific electrical, magnetic, and optical properties, contributing to innovations in electronic devices, solar cells, and more.^5–9^ Recent research has been focused on perovskite materials composed of rare earth (RE) and transition metal (TM) atoms, known as RE-TM-O_3_ perovskites. Substituting some cations (B) or TM elements with magnetic counterparts has led to unexpected magneto-electric and multiferroic properties in these materials.^8,10^ These properties have garnered significant attention due to their potential applications in data storage, spintronics, and next-generation electronic devices. These perovskite substances exhibit both ferromagnetic and ferroelectric ordering, which are interconnected through strain or spin interactions. This unique combination of properties makes them promising candidates for various technological advancements. Researchers have utilized *ab initio* techniques to study the magnetic and electrical characteristics of different perovskites, furthering our understanding of their potential in emerging technologies. As a result, the exploration of RE-TM-O_3_ perovskites has opened up exciting possibilities for innovative materials with multifunctional properties that could revolutionize electronic and magnetic devices.^11–13^ For instance, M. Yaseen *et al.* in 2020 came to the conclusion that the PRCrO_3_ compound's viability for spintronics applications was proven based on its electrical and magnetic properties.^14^ Rashid *et al.* conducted a study using density functional theory (DFT) with the mBJ approximation to investigate the electronic and magnetic properties of the cubic CeCrO_3_ compound in the ferromagnetic (FM) phase.^15^ Their research revealed that CeCrO_3_ exhibits a total magnetic moment of 4.0 *μ*_B_ and possesses half-metallic and ferromagnetic properties. In addition to CeCrO_3_, recent studies have focused on other compounds like CaCrO_3_, BaCrO_3_, and SRCrO_3_, with a particular emphasis on the length of the Cr–O bond.^11^ Materials based on RCrO_3_ compounds demonstrate remarkable adaptability and exceptional physical properties. These materials exhibit characteristics such as the magnetocaloric effect (MCE), exchange bias, and magnetization reversal, making them attractive for various applications. Despite the growing interest in transition metal oxides and their potential applications, there is currently a gap in the literature regarding the physical properties of RCrO_3_ compounds (where X = Ca, Sr, Ba, Hg, *etc.*) for use in electrical or spintronic devices.^16,17^ Understanding the physical properties of these materials is crucial for designing and implementing high-performance devices. Researchers also explore non-equilibrium growth conditions, such as molecular beam epitaxy (MBE) and other physical vapor deposition techniques, to create stable phases of novel materials with tailored properties.

This study aims to explore the structural, thermoelectric, and magnetic properties of HgCrO_3_, a cubic perovskite material, to assess its potential industrial applications. To achieve this, the study utilizes the FP-LAPW-GGA (PBE) approach, a computational method commonly used for electronic structure calculations. One noteworthy aspect of this research is that it appears to be the first of its kind to investigate these specific properties of HgCrO_3_. The lack of existing information on this material highlights the novelty of the study. This pioneering effort to delve into the material's characteristics could potentially serve as a catalyst for further research in this area. Understanding the structural, thermoelectric, and magnetic properties of HgCrO_3_ is essential for unlocking its industrial potential and expanding its application in various fields. This study represents a crucial step toward harnessing the unique properties of HgCrO_3_ for technological advancements and innovations.

## 2. Computational approach

The study focused on evaluating various properties of HgCrO_3_, including its structural, magnetic, elastic, and electronic characteristics. To conduct this assessment, to employed the Density Functional Theory (DFT)^18,19^ framework and utilized the FP-LAPW tool^20^ from WIEN2k,^21^ which is a widely used computational method for electronic structure calculations. One notable aspect of their study is that they incorporated the spin–orbit effect using semi-relativistic techniques, which is essential for accurately modelling the electronic behaviour of materials with heavy elements like mercury (Hg). Understanding these properties is crucial for gaining insights into the behaviour and potential applications of HgCrO_3_. The combination of advanced computational tools and the consideration of the spin–orbit effect allows for a comprehensive analysis of this material. Such studies contribute to our understanding of materials with unique characteristics and can inspire further research in areas where HgCrO_3_ may find practical applications. DFT calculations were used to determine the magnetoelectronic characteristics while taking exchange and correlations into consideration. The structural optimization has been performed employing generalized gradient approximation method (PBE-GGA). For this, the charge convergence requirement of 10–5 has been established as their convergence condition, and the plane wave cut off has been set at 450 eV with a k-grid of 10 × 10 × 10. The convergence criterion for the interaction forces between atoms is set to 0.01 eV Å^−1^. We employed a plane wave cut-off value of *R*_MT_ × *K*_max_ = 7 to obtain convergence in the basis set and discovered the MT radius to be 1.63, 1.54, 1.48 Bohr for Hg, Cr, and O. We increased the radial eigenfunctions of the muffin-tin spheres by spherical harmonics up to *l*_max_ = 10 in order to enhance wave functions within the spheres.^22,23^ Additionally, we increased the interstitial region's potential Fourier charge density up to *G*_max_ = 14. Furthermore, the study extended its focus to explore thermoelectric transport properties, utilizing the quasi-harmonic Debye model and the BoltzTraP algorithm.^24,25^ Due to the fact that it mostly depend on the denser *k*-points, the k-mesh of the Brillouin zone has been increased to 2556 *k*-points. This exploration provides valuable insights into the material's behaviour under different conditions.

## 3. Findings and interpretation

### 3.1 Structural characteristics

**Fig. 1:**
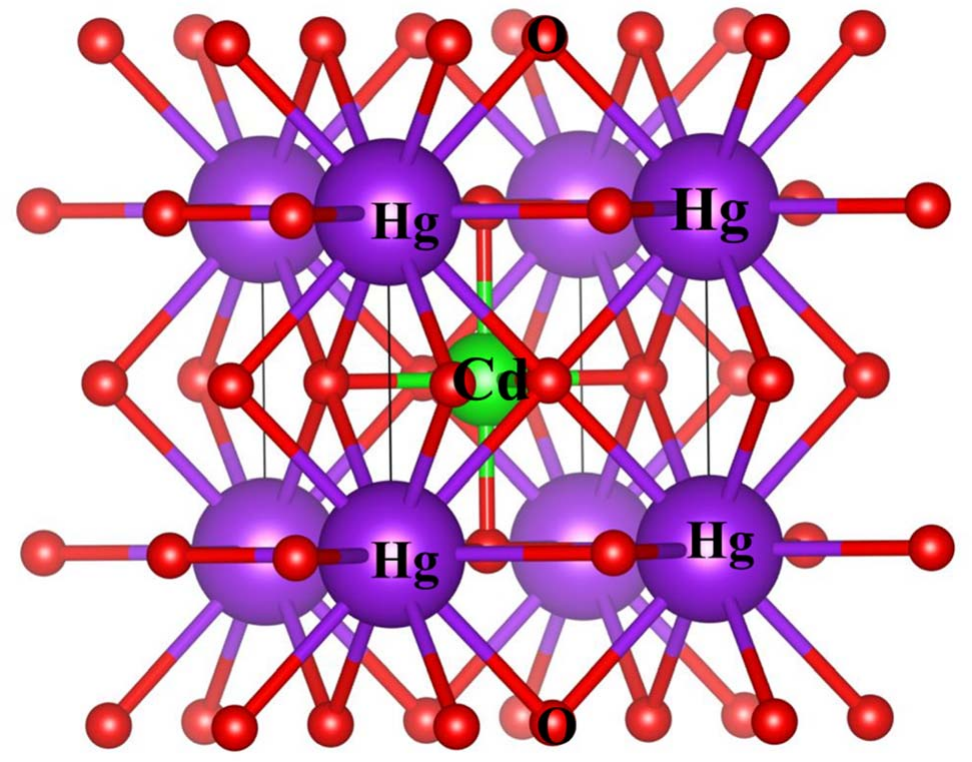
Computed crystal structure of cubic HgCrO_3_.

**Fig. 2:**
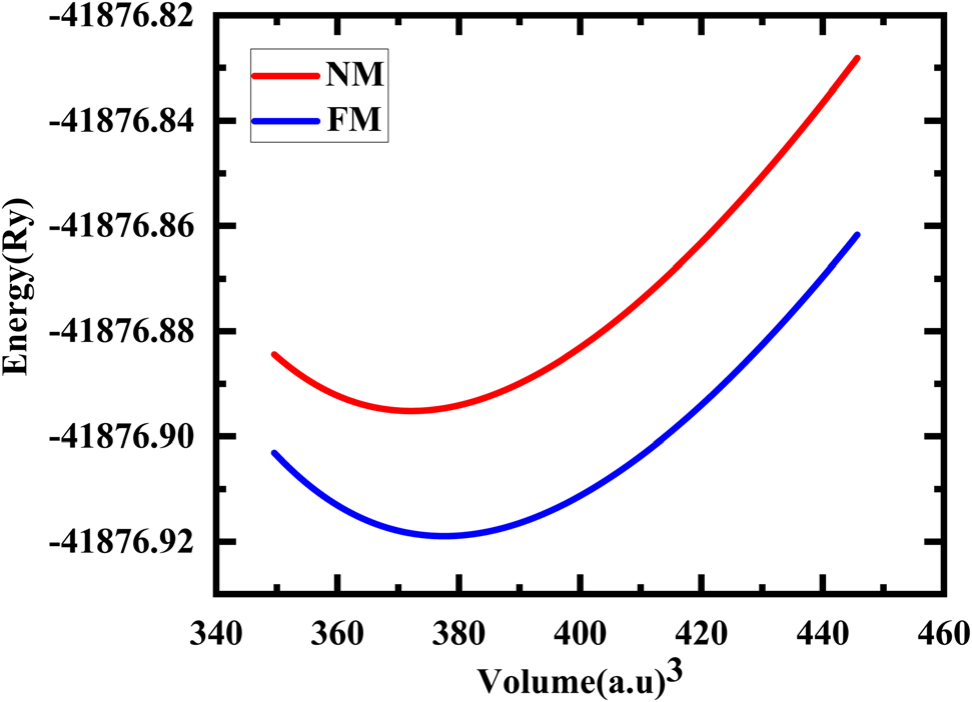
Total energy with the volume optimization curve of HgCrO_3_ in its NM, FM phases.

The study involved an improvement of the structural characteristics of perovskite compounds, focusing on their cubic geometry. Fig. 1 in shows a representation of the fully relaxed structure of these compounds. In this cubic structure, the halide ion O is positioned at coordinates (1/2,1/2,*y*) with *y* equal to 0.249, while the constituent atoms Hg and Cr are located at (0,0,0) and (1/2,1/2,1/2), respectively.^11^ The analysis revealed that these chemicals possess a cubic *Pm*3*m* space group, signifying their structural symmetry. To determine the ideal lattice parameters, we employ the PBE-GGA functional and the Birch Murnaghan equation of state against volume (measured in atomic units) as represented in Fig. 2.^26^ This computational approach allows for precise assessments of the lattice parameters, contributing to a deeper understanding of the material's properties and behaviour in the cubic phase.^27^Table 1 displays the structural optimization's computed results. The computed results are discovered to be consistent with past findings, demonstrating the accuracy of our computation. The tolerance factor (*τ*) to support the cubic structural parameters was also estimated.^23,28,29^ The examined chemical is assumed to have a cubic structure if the indicator parameter's value falls within the range of 0.95 and 1.04. Table 1 displays the anticipated value of the, which indicates the cubic structure. Calculating the cohesive energy and formation energy of the investigated HgCrO_3_ has allowed for the evaluation of its thermodynamic and chemical stabilities,^26,27^ as shown in Table 1. Cohesive energy of the solid is the amount of energy required to form separated neutral atoms in their ground electronic state from the solid. A higher degree of stability is associated with higher cohesive energy levels. The cohesive energy per atom can be calculated using the following formula:1
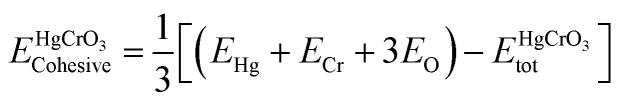
Here 
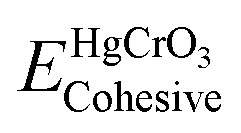
 refers to the total energy of the unit cell, *E*_Hg_, *E*_Cr_, 3*E*_O_ are the total energies of the corresponding elements in their free state of isolated atoms. The computed cohesive energy is 3.05 ev per atom suggesting the chemical stability of the investigated compound. Furthermore formation energy is computed as2
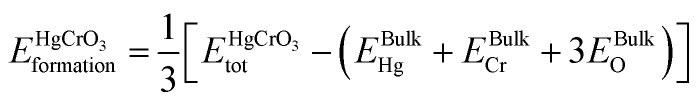

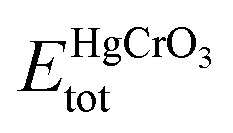
 is the total energy of HgCrO_3_ per formula unit, and *E*^Bulk^_Hg_, *E*^Bulk^_Cr_, 3*E*^Bulk^_O_ are the total energies of Hg, Cr and O in bulk. Table 1 shows that the compound under investigation has a negative formation energy, indicating that it is thermodynamically stability.^30,31^ This stability is essential in understanding the compound's behaviour in various applications, such as in materials science and catalysis. It indicates that HgCrO_3_ is likely to exist and persist in the examined conditions, making it a valuable material for certain applications.

**Table 1:** The calculated lattice parameters *a*_0_, volume *V*_0_, bulk modulus *B*_0_, the derivative of the bulk 
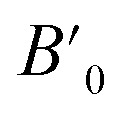
 and equilibrium total energy *E*_0_, cohesive energy (*E*_c_) for HgCrO_3_ for phases in NM and FM states using GGA-PBE approximation

Compound	Phases	*a*_0_ (Å)	*V*_0_ (a^3^)	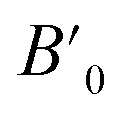	*E*_tot_ (Ry)	*E*_f_ (ev per atom)	*E*_c_ (eV per atom)	Δ*E* (Ry) = *E*_N M_− *E*_FM_
HgCrO_3_	FM	3.82	377.62	190.39	−41876.918940	−2.631	3.05	0.023751
	NM	3.80	372.21	198.99	−41876.895189			
Other^11^		3.76		200.88				

### 3.2 Electronic properties

**Fig. 3:**
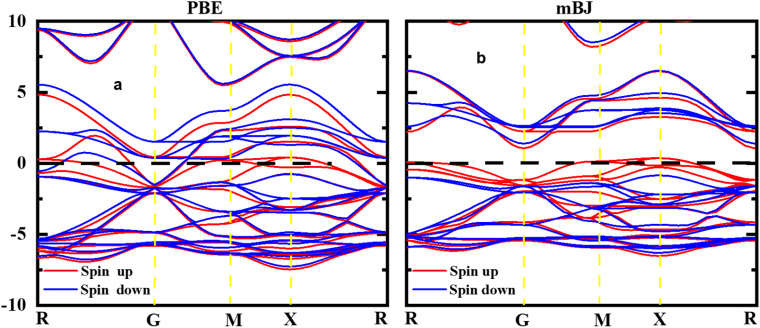
Spin-polarized band structures of perovskite oxide HgCrO_3_ spin up and spin down.

The study of electronic properties is indeed a key component of compounds, crucial for developing materials with specific qualities for various applications. The electronic band structure of a material graphically represents the permissible energy levels for electrons in a solid. This structure is based on how electrons behave in the valence and conduction bands and the gap between them.^32,33^ The size of this energy gap determines whether a material is an insulator, semiconductor, or metal. Moreover, a material's electronic properties, such as electrical conductivity, optical characteristics, and magnetic behaviour's, are largely influenced by the behaviours of electrons in these bands and the width of the gap. In this work, HgCrO_3_, the most stable structure and optimized lattice parameters were likely used to investigate its electrical characteristics.^34,35^ This involves understanding how electrons interact within the material's electronic band structure, which, in turn, affects its overall electronic properties. The electronic band structure of HgCrO_3_ was investigated using Density Functional Theory (DFT), a powerful computational technique for studying the electronic properties of materials. The electronic properties as computed from the GGA reveals the metallic nature of the HgCrO_3_ as represented in Fig. 3(a). However again we considered the mBJ method to endeavour the electronic properties in spin up and spin down. It is observed that bandgap exist both in spin up and spin down, only P-type doping occurs when spin up, which may have better electrical conductivity while spin down shows near-intrinsic type as observed in Fig. 3(b).^12,36^

**Fig. 4:**
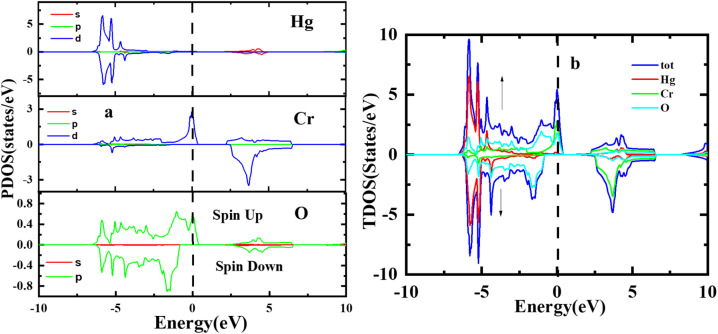
Total and partial density of states of perovskite oxide HgCrO_3_.

Fig. 4 illustrates the computed total and partial densities of states (TDOS and PDOS) for HgCrO_3_. These density of states plots provide critical insights into the electronic structure of the material. TDOS gives an overview of all available electronic states at various energy levels. It helps understand the overall electronic behaviour of HgCrO_3_. PDOS (Partial Density of States) breaks down the electronic states by specifying contributions from individual atoms or orbitals within HgCrO_3_. This allows for a detailed analysis of how specific elements or orbitals affect the electronic properties.^37–39^ The Fermi level, represented by a vertically broken line in the figure, indicates the energy level at which electrons are in their ground state at absolute zero temperature. It's a crucial reference point for understanding the material's conductivity and its classification as a metal, semiconductor, or insulator. Overall, Fig. 4 provides a comprehensive view of the electronic properties of HgCrO_3_, with TDOS, PDOS, and the Fermi level as key components for analysis. The presence of complete Density of States (DOS) profiles above the Fermi level (*E*_f_) provides indicating the prospect of HgCrO_3_ revealing metallic conductivity. In metallic materials, the existence of electronic states available for conduction above the Fermi level is a defining characteristic. A major part of the contribution to the Total Density of States (TDOS) in proximity to the Fermi level comes from the Hg (4d/5d) orbitals. Consequently, it can be inferred that the d orbitals associated with Hg atoms play a central role in facilitating the electrical conductivity of both compounds. The attributes characterizing the electronic states pertaining to Hg's 4d/5d bonding orbitals serve as the principal contributors to the chemical and mechanical stability inherent in HgCrO_3_. These electronic states are instrumental in comprehending the overarching stability exhibited by the material. The TDOS profiles of HgCrO_3_ framework a prevailing presence of Cr-4d/La-5d orbitals, in conjunction with O 5p and Hg 2p states, within the valence and conduction bands of HgCrO_3_, specifically in the energy range spanning from 1.03 eV to 2.25 eV. This elucidates the salient electronic states governing the material's electronic behavior within this energy spectrum. The attractive acknowledges that the Density of States at the Fermi level (*E*_f_, DOSs) wields a pivotal influence on the electrical stability observed in HgCrO_3_ metallic systems. The position of the Fermi level, (*E*_F_), along with its associated strength, bears significant consequences for the phase stability of intermetallic complexes.^40,41^ Notably, complexes characterized by lower *E*_f_ values tend to exhibit heightened stability in comparison to those characterized by higher *E*_f_ values.

### 3.3 Magnetic properties

Magnetism, a fundamental property of materials, finds significant applications in various industries, including electronics, energy, and medicine. When studying a material's magnetic characteristics, one crucial parameter to consider is the magnetic moment, which provides insights into the magnetic field's properties and frequency. This investigation investigates the magnetic spin moments of HgCrO_3_, a complex compound comprising elements such as cadmium (Hg), chromium (Cr), and oxygen (O). To explore its magnetic properties, the total and partial magnetic moments, including polarized spins in the interstitial sites and muffin-tin spheres, were estimated using both the GGA model.^42^ The findings, presented in Table 2, reveal that material exhibit metallic characteristics due to their total magnetic moments per cell unit interestingly, the magnetic moments of Hg and O atoms were found to be relatively modest and had a minimal impact on the overall magnetic moment. Nevertheless, they play a pivotal role in the emergence of magnetism and influence the major band gap's magnitude in HgCrO_3_. This underscores the importance of considering these atoms' magnetic moments when investigating a material's magnetic properties. Furthermore, it was observed that the interstitial region possesses a substantial magnetic moment. This effect can be attributed to a significant charge transfer from Hg and Cr to O, driven by the difference in electronegativity values as represented in Table 2. The high magnetic moment in the interstitial region can have profound implications for the compound's characteristics, including its electrical and optical properties, which are vital for numerous applications.^43^ Furthermore, a noteworthy finding is the substantial magnetic moment observed in the interstitial region. This phenomenon can be attributed to a significant charge transfer mechanism, wherein electrons are transferred from Hg and Cr to O. The underlying explanation lies in the contrasting electronegativity values of these elements. Specifically, oxygen (O) is characterized by a higher electronegativity value of 3.5 on the Pauling scale, while both mercury (Hg) and chromium (Cr) exhibit significantly lower electronegativity values, differing by 1.69 units compared to O. This marked difference in electronegativity levels results in a substantial charge transfer from Hg and Cr to O, consequently giving rise to the magnetic moment observed in the interstitial region. The notable magnetic moment in the interstitial area can wield a profound influence on the compound's overall characteristics, potentially impacting its behavior and properties in various applications.^44^ The compound's electrical and optical properties, for instance, which are crucial for a number of applications, might be impacted by the magnetic moment. The magnetic moment in the interstitial region can also affect the stability and symmetry of the compound's structure.

**Table 2:** The total and atomic magnetic moments of compounds by different XC (PBE-GGA and mBJ-GGA). *M*_tot_ (*μ*_B_): total magnetic moment; *M*_Ti_ (*μ*_B_): Ti magnetic moment; *M*_Ti_ (*μ*_B_): Ti magnetic moment; *M*_Fe_ (*μ*_B_): Fe magnetic moment; *M*_As_ (*μ*_B_): As magnetic moment; *M*_Int_ (*μ*_B_): the magnetic moment in the interstitial region

XC	PBE	mBJ
*M*_interestial_ (*μ*_B_)	0.24	0.27
*M*_Hg_ (*μ*_B_)	0.08	0.02
*M*_Cr_ (*μ*_B_)	1.63	2.06
*M*_O_ (*μ*_B_)	−0.03	−0.12
*M*_tot_ (*μ*_B_)	1.88	2.00

### 3.4 Thermal characteristics

**Fig. 5:**
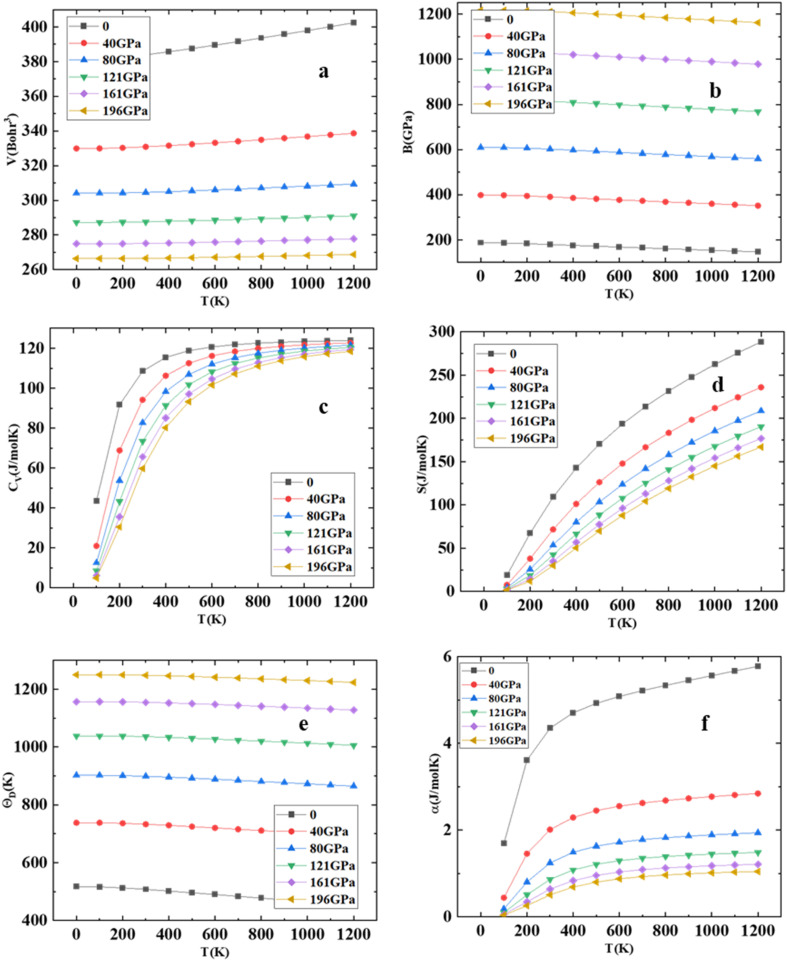
(a) Volume (*V*), (b) Bulk modulus (c) heat capacity at constant volume (*C*_V_), (d) entropy (*S*), (e) Debye temperature (*θ*_D_) and (f) thermal expansion coefficient (*α*) *versus* the different temperatures for HgCrO_3_.

Using Gibbs2 code,^45^ we investigate a comprehensive analysis of the thermodynamic properties of the HgCrO_3_ compound. Our investigation has a temperature range spanning from 0 to 1200 K while considering pressure variations from 0 to 196 GPa, employing the quasi-harmonic model. The variation of volume at diverse pressure and temperature is represented in Fig. 5(a) shows it decreases with increase in temperature. One outstanding finding pertains to the bulk modulus (*B*) of HgCrO_3_ at varying conditions. At 0 K, the bulk modulus reaches its peak value of 210 GPa. This observation signifies that as pressure increases at a given temperature, the bulk modulus also increases while maintaining constant pressure results in a decrease in the bulk modulus as seen in Fig. 5(b). Furthermore, it is noteworthy that the bulk modulus remains relatively constant within the temperature range of 0 to 220 K, after which it gradually decreases with rising temperature. Heat capacity at constant volume (*C*_v_) plays a pivotal role in understanding the vibrational properties of compounds.^23^*C*_v_ exhibits distinct behavior across temperature ranges as seen in Fig. 5(c). At low temperatures below 300 K, *C*_v_ shows a sharp increase with rising temperature, constituting the first phase. Subsequently, in the second phase above 300 K, there is a progressive increase in *C*_v_ with temperature. This intricate behavior of heat capacity provides valuable insights into the compound's thermal characteristics as seen Fig. 5(d) illustrates the temperature-dependent changes in the entropy of HgCrO_3_. The exponential nature of the entropy curves is evident. At a temperature of 300 K and under normal pressure conditions, the entropy of the tested HgCrO_3_ compound is approximately 121.66 J mol^−1^ K^−1^. This data is valuable for evaluating the structural stability of compounds.^23,38^ Additionally, the thermal expansion coefficient, denoted as *α*(*T*), serves as a key indicator of structural stability. Additionally, we computed the Debye temperature (*θ*_D_) for HgCrO_3_, as presented in Fig. 5(e). Notably, the calculated (*θ*_D_) values for HgCrO_3_ are 575 at 0 K and 40 GPa pressure. Linear declines in (*θ*_D_) occur under constant pressure and temperature conditions. It is noteworthy that the effect of pressure serves to elevate the Debye temperature (*θ*_D_)) with increasing pressure and temperature.^46,47^ This comprehensive analysis of thermodynamic characteristics contributes valuable insights that can inform experimental efforts in understanding the behaviour of HgCrO_3_ under varying temperature and pressure conditions. As depicted in Fig. 5(f), exhibit a substantial increase in *α*(*T*) up to 200 K due to the influence of the harmonic component of the Debye model approximation. However, beyond this temperature threshold, the thermal expansion coefficient consistently decreases, highlighting the HgCrO_3_ crystal's remarkable volume invariance.

### 3.5 Transport characteristics

**Fig. 6:**
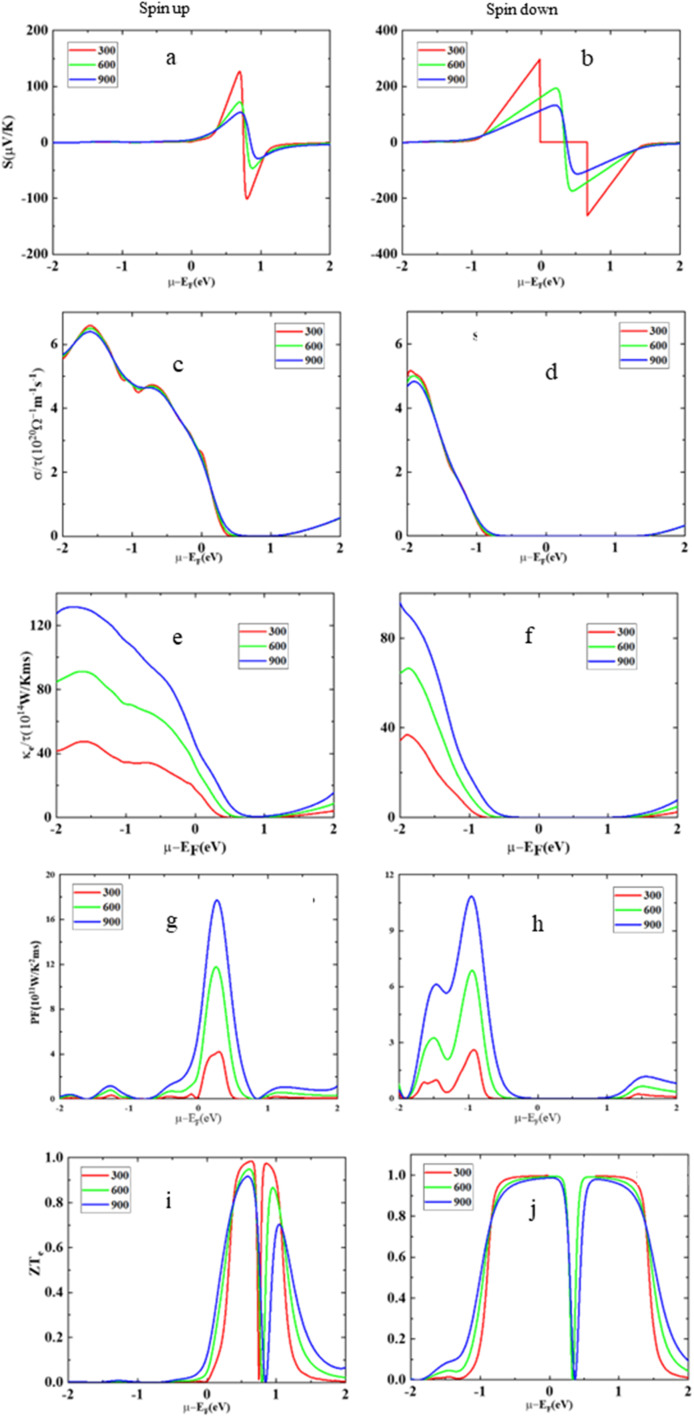
The Seebeck coefficient (*S*) (a) spin-up, (b) spin-down, electrical conductivity (*σ*/*τ*), (c) spin-up, (d) spin-down, electronic thermal conductivity (*κ*_e_/*τ*) (e) spin-up, (f) spin-down, power factor (*S*^2^*σ*) (g) spin-up, and (h) spin-down, figure of merit (*ZT*) (i) spin-up, and (j) spin-down *versus* the chemical potential (*μ*) at different temperatures for HgCrO_3_.

This section of the study focuses on thermoelectric materials, which have significant attention worldwide due to their unique ability to convert thermal energy into usable electricity and *vice versa*. These materials are highly sought after, particularly within the thermoelectric field, because of their dielectric properties and their capability to harness and produce energy. The primary goal of this section is to conduct a comprehensive investigation into various transport coefficients associated with HgCrO_3_. These coefficients are assessed for both the up-spin and down-spin states of the material. The key parameters under scrutiny encompass electrical conductivity (*σ*/*τ*), which provides insights into the material's efficiency in conducting electricity^35^ Seebeck coefficient (*S*), which quantifies a material's ability to generate voltage when exposed to a temperature gradient, thereby facilitating the conversion of heat into electrical energy. Additionally, the figure of merit (*ZT*), a critical metric in the realm of thermoelectric materials, is evaluated to gauge the material's overall thermoelectric performance. It combines information from electrical conductivity, the Seebeck coefficient, and thermal conductivity to assess the material's efficiency in converting heat into electricity. Fig. 6(a and b) is a graphical representation of the variations in the Seebeck coefficient (*S*) for a material called HgCrO_3_ at different temperatures. The Seebeck coefficient is a measure of a material's ability to generate an electric voltage when subjected to a temperature gradient, which is crucial in thermoelectric applications. The key findings *i.e.* up-spin state, when HgCrO_3_ is in the up-spin state (a particular electronic configuration or condition), the Seebeck coefficient remains positive across the entire temperature range. This means that the material consistently generates a positive electric voltage when exposed to temperature differences in this state. The positive value indicates that HgCrO_3_ has a thermoelectric behaviour that is favourable for generating electricity from heat. Spin-down state, In the spin-down state of HgCrO_3_ (another electronic configuration or condition), the Seebeck coefficient increases linearly with temperature throughout the entire temperature spectrum. This suggests that, in this state, as the temperature rises, the material's ability to generate electric voltage also increases in a predictable, linear fashion. This behaviour is essential for understanding how HgCrO_3_ performs in different electronic states and temperature conditions, which is critical information for thermoelectric applications. Overall, this figure provides valuable insights into the thermoelectric properties of HgCrO_3_ under different conditions and temperatures, which can be helpful for designing thermoelectric devices and applications. In Fig. 6(c and d), the data reveals the temperature-dependent behaviour of electrical conductivity (*σ*/*τ*) in HgCrO_3_. Notably, it is important to highlight that the computed electrical conductivity values for the spin-up electron state differ from those obtained for the spin-down electron state, as documented in ref. 45. Specifically, for the spin-up state, the electrical conductivity (*σ*/*τ*) exhibits an increase between temperatures of 300 Kelvin to 900 Kelvin. In contrast, for the spin-down state, there is a decrease in electrical conductivity within the temperature range of 200 Kelvin to 800 Kelvin. The thermal conductivity as represented in Fig. 6(e and f) shows variation with different temperatures. The less thermal conductivity advocates its optimum thermoelectric performance. The power factor reflects the thermoelectric applicability of these compounds as represented in Fig. 6(g and h). Moreover, Fig. 6(i and j) provides an overview of the changes in the figure of merit (*ZT*) across a temperature range spanning from 0 Kelvin to 800 Kelvin, which enables an evaluation of the thermoelectric efficiency of the compound, the discussion highlights that at a temperature of 300 Kelvin, the figure of merit (*ZT*) values for HgCrO_3_ are nearly negligible, indicating limited thermoelectric efficiency at this temperature. However, as the temperature rises, there is a noticeable improvement in the *ZT* values, suggesting that HgCrO_3_ becomes more suitable for thermoelectric applications at higher temperatures.^48^ This observation is significant because it implies that HgCrO_3_ has the potential to be practically utilized in technologies related to energy conversion and utilization, particularly at elevated temperatures where its thermoelectric performance becomes more favourable.

## 4. Conclusions

Comprehensive study, we conducted an in-depth examination of various aspects of HgCrO_3_ perovskite, including its structural, elastic, vibrational, and thermodynamic characteristics. To achieve this, we utilized advanced computational methods, specifically the FP-LAPW (Full-Potential Linearized Augmented Plane Wave) and quasi-harmonic Debye models. We optimized the lattice parameters of HgCrO_3_ using the generalized gradient approximation of the Perdew–Burke and Ernzerhof exchange-correlation functional (GGA-PBE). The results from these optimizations align well with previous research, confirming the accuracy of our calculations. Our findings indicate that both materials under investigation exhibit metallic properties, as evidenced by their electronic energy band structures and overall density of states. Additionally, we identified that the conductivity of both materials is primarily governed by Hg-4d electronic orbitals, further characterizing their electronic behavior. Through the quasi-harmonic Debye model, we delved into several key thermodynamic properties, including the relative Debye temperature, thermal expansion parameter, relative volume, and heat capacity at various temperatures and pressures. Notably, we determined that the heat capacity at constant volume is approximately 52 J mol^−1^ K^−1^. Furthermore, our analysis revealed that the HgCrO_3_ compound demonstrates excellent volume invariance when exposed to high temperatures, as indicated by the coefficient of thermal expansion. It's important to note that this research contributes to a broader theoretical project that encompasses a comprehensive understanding of this molecule, enhancing our knowledge of its characteristics and behaviour.

## Conflicts of interest

There are no conflicts to declare.

## Supplementary Material
